# Normal preoperative levels of gamma-glutamyltransferase predict the absence of common bile duct stones in patients scheduled for laparoscopic cholecystectomy: a retrospective cohort study

**DOI:** 10.1097/MS9.0000000000000281

**Published:** 2023-03-27

**Authors:** Noman Ahmed Khan, Muhammad Imran Siraj, Iqra Anees Rajput, Zahid Ali Memon, Rehan Ramzan Ali, Asna Ursani, Muhammad Muthar Anees, Karan Kumar, Naina, Rahul Robaish Kumar, Rahul Kumar, Ramsha Shahab, Muhammad Sohaib Asghar

**Affiliations:** a Civil Hospital; b Liaquat National Hospital and Medical College; c Liaquat College of Medicine and Dentistry (LCMD); d Jinnah Sindh Medical University; f Jinnah Postgraduate Medical Centre; g Dow University of Health Sciences – Ojha Campus Karachi; e Ghulam Muhammad Mahar Medical College, Sukkur, Pakistan

**Keywords:** choledocholithiasis, gamma-glutamyltransferase (GGT), laparoscopic cholecystectomy, per-operative cholangiogram, symptomatic cholelithiasis

## Abstract

Laparoscopic cholecystectomy, nowadays, is considered the gold standard option for management in patients diagnosed with symptomatic cholelithiasis. Nevertheless, some patients may have coexisting choledocholithiasis, which manifests later in life with grave complications such as cholangitis and pancreatitis. The objective of this study is to evaluate the role of preoperative gamma-glutamyltransferase (GGT) in predicting choledocholithiasis in patients undergoing laparoscopic cholecystectomy.

**Method:**

A total of 360 patients with symptomatic cholelithiasis based on diagnosis aided with abdominal ultrasound were included in the study. The study design was a retrospective cohort. Patients were evaluated based on a comparison between findings of per-operative cholangiogram and laboratory measure of GGT.

**Result:**

The mean age of study participants was 47.22 (±28.41) years. Mean GGT levels were 121.54 (±87.91) U/l. One hundred (27.7%) participants had raised GGT. But only 19.4% had been diagnosed with filling defect positive on cholangiogram. The predictability of GGT for positive cholangiogram is statistically significant at less than 0.001 with an area under the curve of 0.922 (0.887–0.957), sensitivity of 95.7%, specificity of 88.6%, and accuracy of 90%. The standard error reported (0.018) was found to be relatively low.

**Conclusion:**

Based on the provided information, it is concluded that GGT plays an important role in predicting the coexistence of choledocholithiasis in symptomatic cholelithiasis and can be used in the setting where the facility of per-operative cholangiogram is not available.

## Introduction

Highlights

- Gamma-glutamyltransferase (GGT) plays an important role in predicting the coexistence of choledocholithiasis with cholelithiasis.
- It has sensitivity of 95.7%, specificity of 88.6%, and accuracy of 90%.
- Patients can be managed accordingly, and postoperative complications can be avoided.

Laparoscopic cholecystectomy is considered the gold standard management option for patients diagnosed with symptomatic cholelithiasis, and the majority of these cases are managed via the laparoscopic approach nowadays^1^–^3^. Nevertheless, some patients can have concomitant common bile duct (CBD) calculi (incidence 3–33%)^4^,^5^.

These calculi could cause CBD obstruction, leading to abdominal pain, jaundice, and CBD dilatation. However, most patients with concomitant CBD stones usually express no related manifestations, and they are often missed during diagnosis^6^,^7^. This could be due to the small stone size or the floating nature of the stone, which does not yield any obstructive symptoms^8^.

Although most of these CBD stones are asymptomatic, they could lead to serious postcholecystectomy complications. The asymptomatic stone may become impacted after surgery leading to the development of pain, jaundice, or even postoperative biliary leakage. All of these complications negatively impact the postoperative course. It may increase patient morbidity, hospital stay, and healthcare costs^9^,^10^. Thus, it is important to identify patients with silent CBD stones before the operation to avoid such dreadful complications^11^.

Although pelvic-abdominal ultrasonography is the routine radiological imaging method used for patient assessment for biliary stones, this modality is operator-dependent and carries low sensitivity and specificity for CBD stones^12^. Other modalities include magnetic resonance and endoscopic retrograde cholangiopancreatography (MRCP and ERCP)^7^. However, the former has a high cost and is not available in all medical centers^13^,^14^. The latter is considered an invasive procedure, and it is often kept for therapeutic purposes^7^.

Gamma-glutamyltransferase (GGT) is a crucial test used in the laboratory assessment of liver damage and is one of the most routinely requested laboratory assays. GGT is largely synthesized by the mitochondria of hepatocytes, expelled by the biliary tract, and primarily dispersed in intrahepatic bile duct epithelium as well as hepatocyte cytoplasm^15^,^16^.

The current literature does not support the significance of preoperative GGT in predicting the presence of asymptomatic choledocholithiasis in patients with symptomatic cholelithiasis. Therefore, this study was conducted to evaluate the role of GGT in predicting choledocholithiasis in patients who planned to undergo laparoscopic cholecystectomy.

## Method

This study was conducted in the Department of General Surgery in a tertiary care hospital. The study design was a retrospective cohort. Data were collected retrospectively from January 2020 to January 2022. The study protocol is registered with the local registry (UIN: E.R.C-05/2022/46). STROCSS (strengthening the reporting of cohort, cross-sectional and case–control studies in surgery) guidelines were conformed to report the findings of the study^17^. Patients included in the study were those who were diagnosed with symptomatic cholelithiasis and were planned for laparoscopic cholecystectomy. On the other hand, those patients were excluded who presented with obstructive jaundice, cholangitis, hepatic or biliary malignancy, Mirrizi syndrome, acute hepatitis, chronic liver disease, viral hepatitis, uncontrolled systemic comorbidities and dilated CBD on ultrasound. Conditions such as liver steatosis, certain medications, and the use of alcohol are frequent causes for elevated GGT levels, hence the recruiters addressed this during history taking and excluded patients accordingly. Lastly, eight patients who underwent laparoscopic cholecystectomy, which intraoperatively converted into open cholecystectomy were excluded from the present study.

The sensitivity of GGT in the detection of choledocholithiasis in patients undergoing laparoscopic cholecystectomy was 84.1%^4^. The sample size was calculated using the WHO sample size calculator. A minimal sample size of 187 was required to achieve a 95% CI that the real value is within ±5% of the measured value.

All cases were dependent upon history taking and local and general abdominal examination. The diagnosis of cholelithiasis was confirmed on transabdominal ultrasound. The values of preoperative GGT were assessed for the patients undergoing laparoscopic cholecystectomy for symptomatic cholelithiasis. GTT levels were measured 1 day before cholecystectomy. If multiple determinations were done, GGT levels on the admission day of the hospital were taken into consideration. Intraoperative cholangiograms were performed in all patients included in the study, and their findings were correlated with the levels of GGT. The cutoff laboratory value of GGT for males is 49 and 32 U/l for females. Tests are performed on a fully computerized EMP-168 autoanalyzer, which utilizes a semiautomated method to measure GGT levels.

SPSS software (version 25.0) was used to code and analyze the data gathered. The *χ* ^2^ test was used to compare the outcome. *P* values less than 0.05 were considered significant for all the applied tests. Receiver operating characteristic analysis was used to obtain diagnostic accuracy.

## Result

The mean age of study participants was 47.22 (±28.41) years. Mean GGT levels were 121.54 (±87.91) U/l. The baseline characteristics of the study population are given in Table 1.

**Table 1:** Baseline characteristics of the study population (*n*=360).

Variables	Characteristics	Frequency, *N* (%)
Gender	Male	110 (30.6)
	Female	250 (69.4)
Comorbidities	Diabetes	30 (8.3)
	Hypertension	48 (13.3)
Clinical characteristics	Filling defect positive on cholangiogram	70 (19.4)
	GGT raised	100 (27.7)

GGT, gamma-glutamyltransferase.

Approximately 27.7% of participants had raised GGT. But only 19.4% had been diagnosed with filling defect positive on cholangiogram. Table 2 showcased the cross-tabulation of raised GGT with positive intraoperative cholangiogram.

**Table 2:** Cross-tabulation of raised GGT with positive intraoperative cholangiogram.

	GGT raised, *N* (%)	GGT not raised, *N* (%)
Intraoperative cholangiogram		
Positive	67 (67.0)	3 (1.2)
Negative	33 (33.0)	257 (98.8)
*P* value	<0.001	
Sensitivity	95.7%	
Specificity	88.6%	
Positive predictive value	67.0%	
Negative predictive value	98.8%	
Positive likelihood ratio	8.41	
Negative likelihood ratio	0.05	
Accuracy	90.0%	

GGT, gamma-glutamyltransferase.

**Figure 1:**
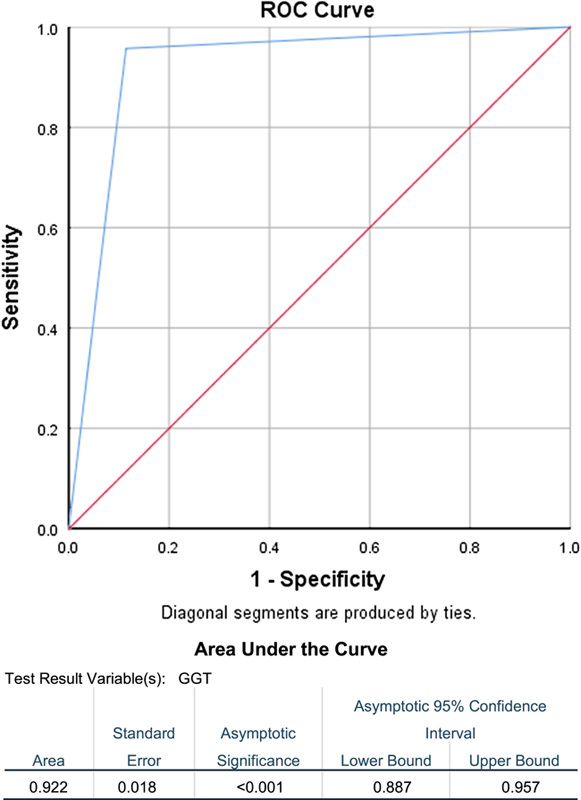
Receiver operating characteristic statistics for area under the curve of raised gamma-glutamyltransferase for positive cholangiogram.

The predictability of GGT for positive cholangiogram is statistically significant at less than 0.001 with an area under the curve of 0.922 (0.887–0.957), sensitivity of 95.7%, specificity of 88.6%, and accuracy of 90% as shown in Figure 1. The standard error reported for this analysis is 0.018, which is relatively low. The positive likelihood ratio (LR) of 8.41 and negative LR of 0.05 was concluded.

## Discussion

Preoperative evaluation of asymptomatic choledocholithiasis with symptomatic cholelithiasis is an important prospect in the management of patients undergoing laparoscopic cholecystectomy. Operating in the presence of missed CBD stones may increase postoperative complications like jaundice, bile leakage, cholangitis, or pancreatitis^18^. MRCP is a valid and accurate radiological modality for the assessment of CBD, as it has sensitivity and specificity of 95% and 97%, respectively, in determining the presence of obstruction and its level^13^. Although we depend on this modality as the gold standard for diagnosing CBD stones, it has an expensive cost and is not available in all settings^19^. Other available options for diagnosing CBD stones are ERCP, intraoperative cholangiogram, and endoscopic ultrasound^20^–^22^. ERCP is preferred as a therapeutic option as it could lead to severe complications like pancreatitis^23^. Intraoperative cholangiogram could elongate procedure time by about 20 min^24^, and it has a high false-positive rate reaching up to 67%, leading to unnecessary ERCP or CBD exploration^24^,^25^. Endoscopic ultrasound also is an expensive procedure that needs general anesthesia, an expert physician, and high-care facilities^26^.

Yang *et al*.^4^ reported that GGT had a sensitivity and specificity of 84.1% and 72%, respectively, in detecting CBD stones in patients undergoing cholecystectomy. In the study conducted by Jovanović *et al*.^27^, GGT was the only significant predictor of CBD stones among all of the tested biochemical markers. Both alkaline phosphatase and GGT had been studied for predicting the presence of CBD stones, with conflicting results for GGT. Alkaline phosphatase was already reported widely and commonly used in this context, for instance, developing predictive models for clinical suspicion of CBD stones^28^. However, we only focused on GGT elevation in the light of detecting CBD stones, unlike another cohort study which determined the accuracy of these markers in the earliest stages of acute biliary pancreatitis^29^. GGT was found more significant (*P*<0.001) than alkaline phosphatase (*P*=0.028) per 10 units increase in their prediction. Our results reported high sensitivities, specificities, and accuracies, together with positive and negative predictive values and area under the receiver operating characteristic curve of 0.922. However, positive and negative predictive values observed in this study do not apply to other studies where the prevalence of abnormality may differ. To strengthen this reporting, we have also reported LRs to supplement the sensitivity and specificity values. High levels of GGT and alkaline phosphatase can also suggest the spontaneous passage of stones from CBD, as suggested by another retrospective study^30^. However, the usefulness of these markers in the postoperative course is not significant^31^.

Data collection done in this study was from a single tertiary care hospital, and the sample size was relatively small. There are certain drawbacks to this study. A high false-positive rate would lead to reaching a sensitivity and specificity that are suboptimal and limited. This is a relevant concern and should be stressed and discussed in future studies. Multicentric studies should be conducted to evaluate and devise a better management plan for the patient to avoid postoperative complications. The positive predictive value of elevated GGT levels was 67%, which implies that one-third of patients with elevated levels will not have evidence for the presence of CBD stones. This is a marked limitation. However, the negative predictive value may be more interesting since this is exceptionally high: 98%. This means that when preoperative GGT levels are normal, the presence of CBD stones is highly unlikely. Finally, we only analyzed the predictive value of GGT, but other common laboratory parameters such as alkaline phosphatase, aspartate aminotransferase, and/or alanine aminotransferase can be explored in the follow-up studies.

## Conclusion

Based on the above-mentioned findings, it can be concluded that preoperative GGT should be evaluated in a patient with symptomatic cholelithiasis undergoing laparoscopic cholecystectomy to diagnose asymptomatic choledocholithiasis so that patients can be managed accordingly and postoperative complications can be avoided.

## Ethical approval

Ethical approval was taken in this study from the institutional review board of Liaquat National Hospital (Ref:App.#E.R.C-05/2022/46).

## Patient consent

Consent to participate from the patients was waived and not required due to the retrospective nature of the data collection.

## Sources of funding

The authors declare that they have no commercial associations (e.g. consultancies, stock ownership, equity interest, patent/licensing arrangement, etc.) or funding with this article.

## Author contribution

N.A.K. and M.S.A.: conceived the idea; M.I.S., I.A.R., R.R.A., A.U., and M.M.A.: collected the data; K.K., N., and R.R.K.: analyzed and interpreted the data; N.A.K., M.S.A., and M.I.S.: write-up of the manuscript; R.S., Z.A.M., and R.K.: reviewed and revised the manuscript for intellectual content critically. All authors approved the final version of the manuscript.

## Conflicts of interest

The authors declare that there are no conflicts of interest.

## Research registration unique identifying number (UIN)

1. Name of the registry: Liaquat National Hospital.
2. Unique identifying number or registration ID: (UIN: E.R.C-05/2022/46).
3. Hyperlink to your specific registration (must be publicly accessible and will be checked): none.

## Guarantor

Muhammad Sohaib Asghar.

## Data availability statement

Data can be made available on request from the corresponding author.

## Provenance and peer review

Not commissioned, externally peer-reviewed.
